# Supplementary material for: Lactobacillus helveticus Isolated from Raw Milk Improves Liver Function, Hepatic Steatosis, and Lipid Metabolism in Non-Alcoholic Fatty Liver Disease Mouse Model
Source: Microorganisms. 2023 Sep 30;11(10):2466. doi: 10.3390/microorganisms11102466 (PMC10609090; doi:10.3390/microorganisms11102466)
Supplement: Supplementary file 1 [file microorganisms-11-02466-s001.zip › microorganisms-2624106-supplementary.pdf]

Table S1. Ingredient composition of NAFLD-induced diet fed to mice

Ingredients	Grams
Casein, Lactic, 30 Mesh	200.00 g
Cystine, L	3.00 g
Fructose	200.00 g
Sucrose, Fine Granulated	100.00 g
Lodex 10	100.00 g
Solka Floc, FCC200	50.00 g
Palm Oil, Bleached, Deodorized	135.00 g
Soybean Oil, USP	25.00 g
Lard	20.00 g
Mineral mix S10026B	50.00 g
Choline Bitartrate	2.00 g
Vitamin mix V10001C	1.00 g
Cholesterol, NF	18.00 g
Dye, Blue FD&C #1	0.03 g
Dye, Red FD&C #40	0.03 g
Total	904.05 g

Table S2. List of lactic acid bacteria strains isolated from raw milk.

Samples name	Strains
LPC16	*Lacticaseibacillus paracasei* LPC16
LPC46	*Lacticaseibacillus paracasei* LPC46
LPC62	*Lacticaseibacillus paracasei* LPC62
LF150	*Limosilactobacillus fermentum* LF150
LP153	*Lactiplantibacillus plantarum* LP153
LP156	*Lactiplantibacillus plantarum* LP156
LP157	*Lactiplantibacillus plantarum* LP157
LP158	*Lactiplantibacillus plantarum* LP158
LP161	*Lactiplantibacillus plantarum* LP161
LR162	*Limosilactobacillus reuteri* LR162
LP164	*Lactiplantibacillus plantarum* LP164
LP172	*Lactiplantibacillus plantarum* LP172
LP173	*Lactiplantibacillus plantarum* LP173
LP174	*Lactiplantibacillus plantarum* LP174
LP175	*Lactiplantibacillus plantarum* LP175
LP176	*Lactiplantibacillus plantarum* LP176
LP177	*Lactiplantibacillus plantarum* LP177
LP178	*Lactiplantibacillus plantarum* LP178
LP194	*Lactiplantibacillus plantarum* LP194
LP197	*Lactiplantibacillus plantarum* LP197
HY7804	*Lactobacillus helveticus* HY7804
LR205	*Limosilactobacillus reuteri* LR205
LRH216	*Lacticaseibacillus rhamnosus* LRH216
LPC226	*Lacticaseibacillus paracasei* LPC226
LP238	*Lactiplantibacillus plantarum* LP238
LS246	*Ligilactobacillus salivarius* LS246

LS248	*Ligilactobacillus salivarius* LS248

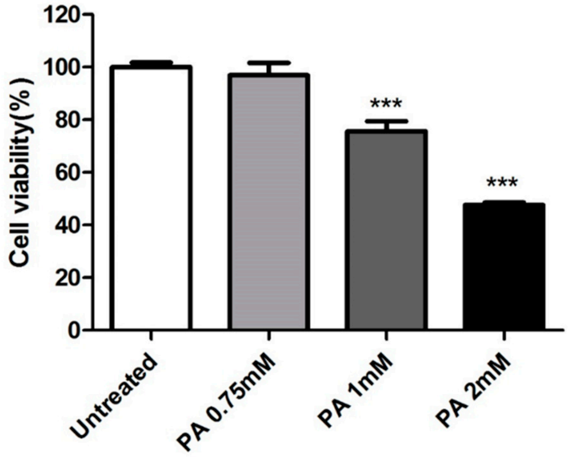

Figure S1. Viability of HepG2 cells treated with palmitic acid.

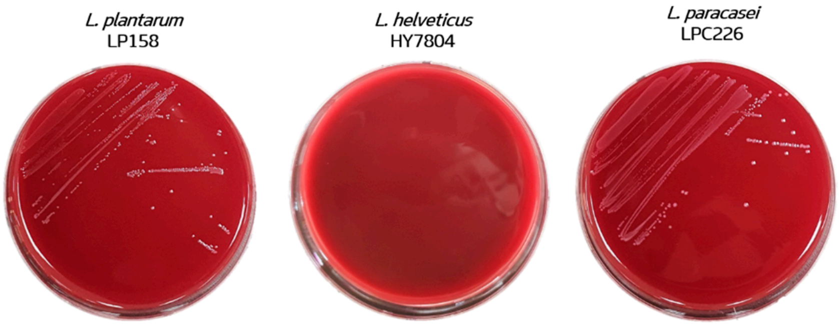

Figure S2. Hemolytic activity of LP158, HY7804 and LPC226 in blood agar plates.

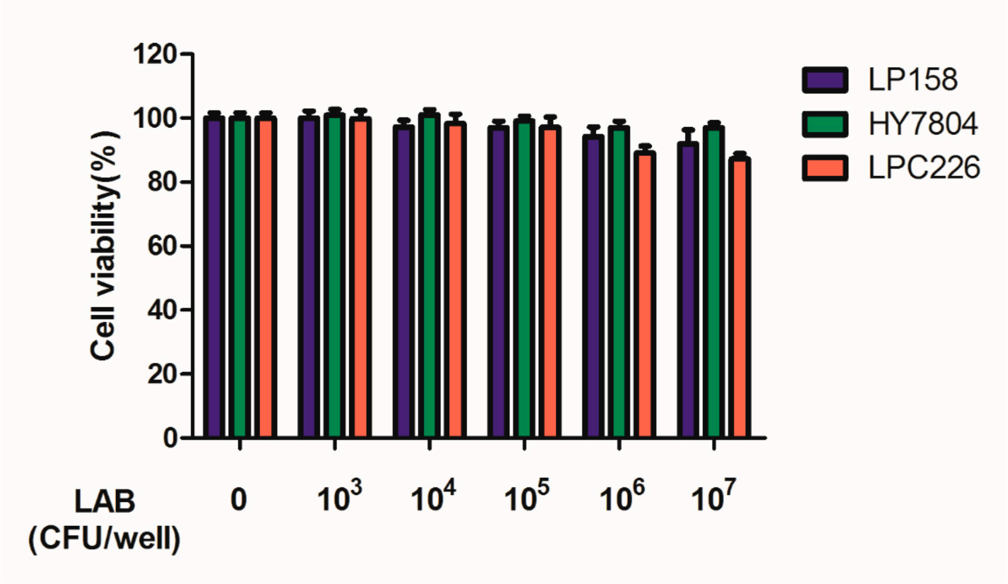

Figure S3. Viability of HepG2 cells treated to LP158, HY7804, and LPC226. LAB, Lactic acid bacteria
